# miR-497-5p inhibits gastric cancer cell proliferation and growth through targeting PDK3

**DOI:** 10.1042/BSR20190654

**Published:** 2019-09-06

**Authors:** Lan Feng, Kai Cheng, Rongjia Zang, Qingdong Wang, Jianjie Wang

**Affiliations:** 1 Department of Infection Medicine, First Affiliated Hospital of Jiamusi University, Jiamusi, Heilongjiang Province, People’s Republic of China; 2 Department of Immunology, Basic Medical College, Jiamusi University, Jiamusi, Heilongjiang Province, People’s Republic of China

**Keywords:** apoptosis, cell cycle, DNA synthesis, gastric cancer, miR-497-5p, PDK3

## Abstract

MicroRNA plays an important role in gastric cancer (GC) development, while the function of miR-497-5p in this disease remains unknown. In the present study, we demonstrated miR-497-5p as a tumor suppressive microRNA in GC. miR-497-5p was down-regulated in GC tissues and its expression was associated with the disease stage. Inhibition of miR-497-5p promoted GC cell proliferation and growth. By contrast, miR-497-5p ectopic expression suppressed the proliferation and growth of GC cells. In addition, miR-497-5p inhibited DNA synthesis and enhanced apoptosis in GC cells. The cell cycle progression was suppressed by miR-497-5p. Mechanistically, miR-497-5p directly targeted and suppressed the expression of pyruvate dehydrogenase kinase 3 (PDK3), which is highly expressed in GC tissues. Over-expression of PDK3 promoted the proliferation of GC cells. Our study revealed that miR-497-5p inhibited GC cell proliferation and growth via targeting PDK3.

## Introduction

Gastric cancer (GC) is one of the most common cancers at Eastern Asia and the second cause of malignancy-related death throughout the world [1]. Although a large amount of patients benefit from the surgical and adjuvant treatment strategies, their 5-year survival rate remains still low and the relapse rate is pretty high. Understanding the pathological and molecular events of gastric cancer development is critical to cure this malignancy.

MicroRNAs (miRNAs) are non-coding RNA molecules of 16–24 nucleotides that negatively regulate the expression of their target genes at the post-transcriptional level [2,3]. Dys-regulation of miRNAs has been shown to participate in the carcinogenesis and metastasis of gastric cancer. For example, down-regulated microRNA-490-3p serves as a tumor suppressor in gastric cancer. It inhibits the cell proliferation and induces the apoptosis of gastric cancer cells via direct targeting AKT1 [4]. miR-5590-3p suppresses the gastric tumorigenesis by negatively regulating DDX5/AKT/m-TOR pathway [5]. Recently, some studies have shown that miR-497-5p functions as a tumor suppressive miRNA in various cancers, including melanoma [6], osteosarcoma [7] and angiosarcoma [8]. The hTERT, ADP ribosylation factor-like protein 2 and KCa3.1 are potential target of miR-497-5p in these cancers. However, the role of miR-497-5p and its downstream target in gastric cancer remain unknown.

PDK3 is a member of the pyruvate dehydrogenase kinase (PDK) family that regulates the metabolic switch in cancer cells [9–11]. It is over-expressed in colon cancer tissues and enhances the drug resistance of colon cancer cells under hypoxia condition [12]. Furthermore, the oncogenic role of PDK3 is also found in other cancer types, such as acute myeloid leukemia [13], glioblastoma [14] and lung cancer [15]. Nevertheless, the involvement of PDK3 in gastric cancer remains to be determined.

In the present study, we aimed to investigate the role of miR-497-5p in GC. Down-regulation of miR-497-5p was observed in gastric cancer tissues based on our results and the TCGA database. miR-497-5p over-expression suppressed the proliferation and colony growth of GC cells, while inverse results were found in GC cells with reduced miR-497-5p expression. Moreover, miR-497-5p directly targeted PDK3 at the 3′UTR region. Ectopic expression or knockdown of PDK3 reversed the phenotypes regulated by miR-497-5p. Our study proposed that miR-497-5p was a tumor suppressive microRNA in GC.

## Materials and methods

### Gastric cancer tissues

All the GC patients were enrolled between 2016 and 2018 at the First Affiliated Hospital of Jiamusi University. The research has been carried out in accordance with the World Medical Association Declaration of Helsinki, and that all subjects provided written informed consent. The present study was also supported by the Clinical Research Ethics Committee of the First Affiliated Hospital of Jiamusi University. All of the tissues were collected before any therapeutic intervention. The age of the patients was between 53 and 65 years old. The patients were divided into TMNII (*n* = 6) and TMNIV (*n* = 9) stage by three independent pathologists. The GC tissues and normal tissues, and the cancer tissues of stage TMNII and TMNIV were subjected to quantitative real-time PCR (qRT-PCR) analysis of miR-497-5p.

### TCGA database analysis

The transcript of miR-497-5p and PDK3 in GC patients was analyzed from the websites of The Cancer Genome Atlas (http://cancergenome.nih.gov).

### Cell culture

GC cells SGC7901 and AGS were purchased from American Type Culture Collection (Manassas, VA, USA). All the cells were cultured in Dulbecco modified Eagle’s medium (DMEM) (Corning), supplied with 10% FBS and 1% penicillin/streptomycin solution. The cell culture was maintained in a 37°C incubator with 5% CO_2_.

### Oligonucleotide transfection

miR-497-5p mimics and mimics control (including miR-497-5p agomir and its control), miR-497-5p inhibitors and inhibitors control (including antagomir and its control) were synthesized from RiboBio company. Oligonucleotide transfection was conducted using lipofectamine 2000 reagent (Invitrogen), following the manufacturer’s protocols. The efficacy was assessed by qRT-PCR assay.

### Lentivirus-mediated PDK3 over-expression assay

The coding sequence of PDK3 was cloned into the pCDH lentivirus vectors. Then empty and PDK3-cloned pCDH vectors were co-transfected with the packaging vectors PSPAX2 and PDM2G into 293T cells. 72 h later, the virus supernatants were harvested and filtered through the 0.45 μm filters. Then the Ctrl and PDK3 lentivirus were subjected to the infection of indicated cells.

### RNA interference

siRNA against PDK3 were obtained from GenePharma company. siCtrl or siPDK3 oligonucleotides were transfected into indicated cells at the concentration of 100 nM by Lipofectamine 2000 (Invitrogen), following to the manufacturer’s protocols. The target sequences of PDK3 were GCCGCTCTCCATCAAACAA.

### RNA extraction and quantitative real-time PCR

Total RNA was extracted from GC cells by TRIzol reagent (Invitrogen, USA). The RNA was qualified by Agarose gel electrophoresis

For microRNA quantification, the reverse transcription was performed using High Capacity RNA-to-cDNA kit. qRT-PCR was then determined by TaqMan probe (Roche). The miR-497-5p abundance was measured with the TaqMan probe and Mater Mix (Thermo Fisher Scientific). U6 serves as internal control.

For mRNA quantification, equal amount of total RNA was subjected to reversed transcription using ReverTra Ace® qPCR RT Master Mix (TOYOBO, Japan). Quantitative real-time PCR experiments were conducted using TransStart Green qPCR SuperMix (TransGen Biotech, Beijing, China) on a Bio-rad IQ 5 machine. The PCR primer sequences were as follow: PDK3 forward, 5′-CGCTCTCCATCAAACAATTCCT-3′, and reverse, 5′-CCACTGAAGGGCGGTTAAGTA-3′; GAPDH forward: 5′-TGACTTCAACAGCGACACCCA-3′, and reverse: 5′-CACCCTGTTGCTGTAGCCAAA-3′. GAPDH serves as internal control.

### Western blot assays

Total proteins were extracted from SGC7901 cells using RIPA buffer (Beyotime). Equal amount of the proteins were separated on the odium dodecyl sulfate polyacrylamide gel electrophoresis (SDS-PAGE), followed by transferring to PVDF membranes. Then the membranes were blocked with 5% skimmed milk at room temperature for 60 min, and incubated with primary antibodies (caspase 3, caspase 9, PDK3 and β-actin) at 4°C overnight. After washing by PBST for three times, the membranes were incubated with HRP-conjugated secondary antibodies. Subsequently, they were subjected to chemiluminescence analysis using the ECL-Plus kit (Amersham Biosciences). Antibodies against caspase 3, caspase 9 and PDK3 were from Cell Signaling. Antibody against β-actin and all the secondary antibodies were from Santa Cruz.

### CCK assay

The viability of GC cells was detected by CCK assay. Briefly, the SGC7901 and AGS cells were transfected with NC and miR-497-5p mimics, or were transfected with NC and miR-497-5p inhibitors. A total of 3000 SGC7901 and AGS cells containing 200 μl culture medium were seeded in 96-well plates. 1, 2, 3 and 4 days later, 20 μl CCK buffer was added into each well and the plates were incubated at 37°C for 2 h. Then optical density (OD) value of 450 nm was measured on a micro-plate reader.

### Colony formation assay

Equal number of SGC7901 and AGS transfected with NC and miR-497-5p agomir (durable mimics), or transfected with NC and miR-497-5p antagomir (durable inhibitors) were seeded and cultured at 37°C. 6–9 days later, the culture medium was removed and the plates were washed by PBS. Then the plates were fixed with methanol for half an hour and subjected to staining by crystal violet solution. The colony images were photographed by Nikon camera.

### EDU staining

EDU staining was performed using BeyoClick™ EdU Cell Proliferation Kit with Alexa Fluor 555, following to the manufacturer’s instructions. In brief, a total of 3×10^5^ SGC7901 and AGS cells were seeded on the coverslips in six-well plates. Eighteen hours later, the cells were incubated with EDU for 2–4 h at 37°C. Then they were washed by PBS for three times and fixed with 4% paraformaldehyde. After incubating with 0.3% Triton X-100, the cells were stained with Click Addictive Solution and DAPI. The images were collected under the fluorescence microscope.

### Caspase 3/caspase 7 activity measurement

SGC7901 cells were seeded into the 96-well plates. A total of 100 μl caspase-Glo reagent (Promega), which was prepared following the manufacturer’s instructions, was added into each well. After rotating at 300–500 rpm for 30 min, the plates were incubated at room temperature for 90 min. The activity of caspase 3/caspase 7 was measured on the microplate reader.

### Cell cycle detection

Propidium iodide (PI) staining and flow cytometry were used to analyze the cell cycle distribution. Briefly, indicated GC cells were seeded in six-well plates. The cells were stained with PI and the PI absorbance was detected by flow cytometry.

### Apoptosis analysis

The apoptosis of GC cells was detected using Annexin-V-APC kit (Ebioscience, USA), following the manufacturer’s instructions. Indicated cells were washed by PBS and re-suspended by staining buffer. Annexin V-APC regent was added into the cell suspension. After incubation at room temperature for 15 min, apoptosis was measured on the flow cytometry.

### Luciferase reporter assay

The WT or MU 3′UTR sequences of PDK3 were cloned into the psi-CHECK vectors (Promega). The vectors were described previously [16]. Transfection was performed with Lipofectamine 2000 (Invitrogen) in SGC7901 cells, according the manufacturer’s protocols. Dual-Luciferase Reporter Assay System (Promega) was used to detect the luciferase activity, which was measured on a luminometer (Berthold Technologies). Firefly luciferase activity was normalized to Renilla luciferase activity.

### Immunohistochemistry staining

GC and normal tissues were fixed in 4% formalin. Paraffin-embedded sections were de-paraffinized in xylene and hydrated in graded alcohol. Antigen retrieval was conducted with citrate buffer. Endogenous peroxidase blockage was performed in 3% hydrogen peroxide. The slides were blocked with 10% goat serum and incubated with PDK3 primary antibody at 4°C overnight. The images were photographed using the microscope.

### Statistical analysis

GraphPad prism was used to analyze the data. Un-paired student’s *t* tests were applied to analyze the difference between two groups. *P* value of less than 0.05 was considered statistically significant.

## Results

### miR-497-5p is down-regulated in GC tissues and cells

**Figure 1:**
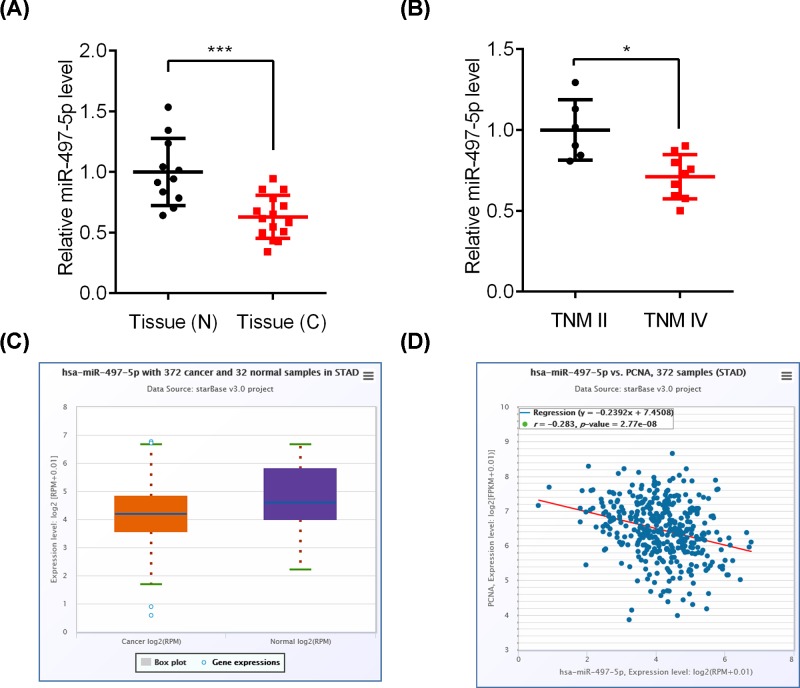
miR-497-5p is down-regulated in GC patients (**A**) qRT-PCR analysis of miR-497-5p in GC (*n* = 15) and normal tissues (*n* = 11). ***P<0.001. (**B**) miR-497-5p expression in GC tissues of stage TMNII (*n* = 6) and TMNIV (*n* = 9). *P<0.05. (**C**) miR-497-5p expression in stomach adenocarcinoma (*n* = 372) and normal tissues (*n* = 32) that was analyzed from TCGA database. *P*<0.001. (**D**) Correlation between miR-497-5p and PCNA mRNA expression in stomach adenocarcinoma tissues (*n* = 372) that was analyzed from TCGA database. *P*<0.001.

In order to investigate the significance of miR-497-5p in GC patients, we measured the miR-497-5p expression in GC tissues and normal tissues. qRT-PCR results showed that the expression of miR-497-5p was reduced in GC tissues as compared with normal tissues (Figure 1A). Then the GC patients were divided into TMNII and TMNIV stage. miR-497-5p level was relatively lower in the cancer samples of TMNIV stage than those of TMNII stage (Figure 1B). We also collected the information of miR-497-5p expression from the TCGA database and consistent results were observed (Figure 1C). Furthermore, miR-497-5p was negatively correlated with the expression of cancer marker PCNA in GC patients based on the TCGA database (Figure 1D). Our results suggested that miR-497-5p might be involved in GC development.

### miR-497-5p suppresses the proliferation of GC cells

**Figure 2:**
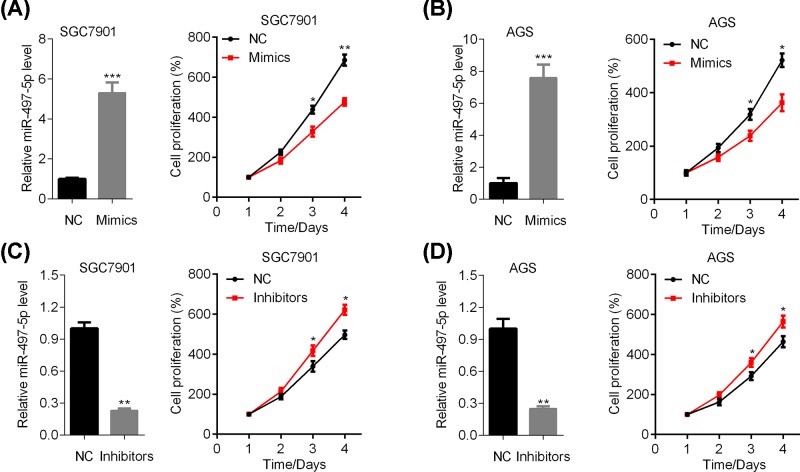
miR-497-5p represses the proliferation of GC cells (**A,B**) Scrambled miRNA (NC) or miR-497-5p mimics transfected SGC7901 (A) and AGS (B) cells were subjected to qRT-PCR analysis of miR-497-5p and CCK analysis of cell proliferation. *P<0.05, **P<0.01, ***P<0.001. (**C,D**) qRT-PCR results of miR-497-5p and CCK analysis of cell viability in scrambled miRNA (NC) or miR-497-5p inhibitors transfected SGC7901 (C) and AGS (D) cells. *P<0.05, **P<0.01.

To explore the role of miR-497-5p in GC cell growth, we transfected the SGC7901 and AGS cells with scrambled miRNA (NC) or miR-497-5p mimics. Then these cells were subjected to CCK assay of proliferation. miR-497-5p over-expression inhibited the growth of both SGC-7901 and AGS cells (Figure 2A,B). Furthermore, SGC7901 and AGS cells were transfected with scrambled miRNA (NC) or miR-497-5p inhibitors and the viability was analyzed by CCK assay. By contrast, miR-497-5p down-regulation promoted the proliferation of both SGC7901 and AGS cells (Figure 2C,D). Taken together, miR-497-5p suppresses the growth of GC cells.

### miR-497-5p inhibits the colony formation of GC cells

**Figure 3:**
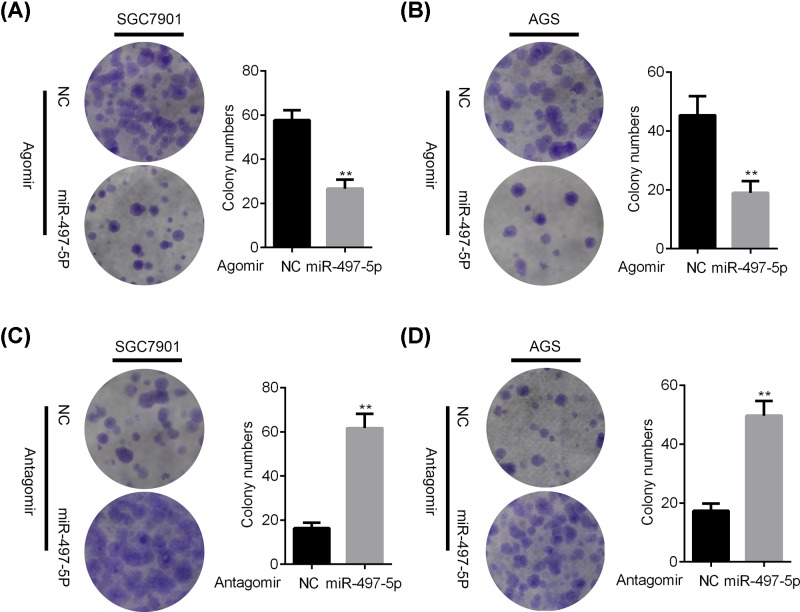
miR-497-5p suppresses the colony formation of GC cells (**A,B**) NC or miR-497-5p agomir transfected SGC7901 (A) and AGS (B) cells were subjected to colony formation assay. **P<0.01. (**C,D**) NC or miR-497-5p antagomir transfected SGC7901 (C) and AGS (D) cells were subjected to colony formation assay. *P<0.05, **P<0.01.

We then investigated the effect of miR-497-5p on GC cell colony formation. Durable microRNA mimics or inhibitors (agomir or antagomir) were used to transfect SGC7901 and AGS cells, followed by colony growth analysis in these cells. In consistent with the CCK results, miR-497-5p agomirs suppressed the colony formation, while the antagomirs promoted the colony growth of SGC7901 and AGS cells (Figure 3A–D). Our study suggested that miR-497-5p functioned as a tumor suppressive microRNA in GC.

### miR-497-5p reduces DNA synthesis and regulates apoptosis-related gene expression in GC cells

**Figure 4:**
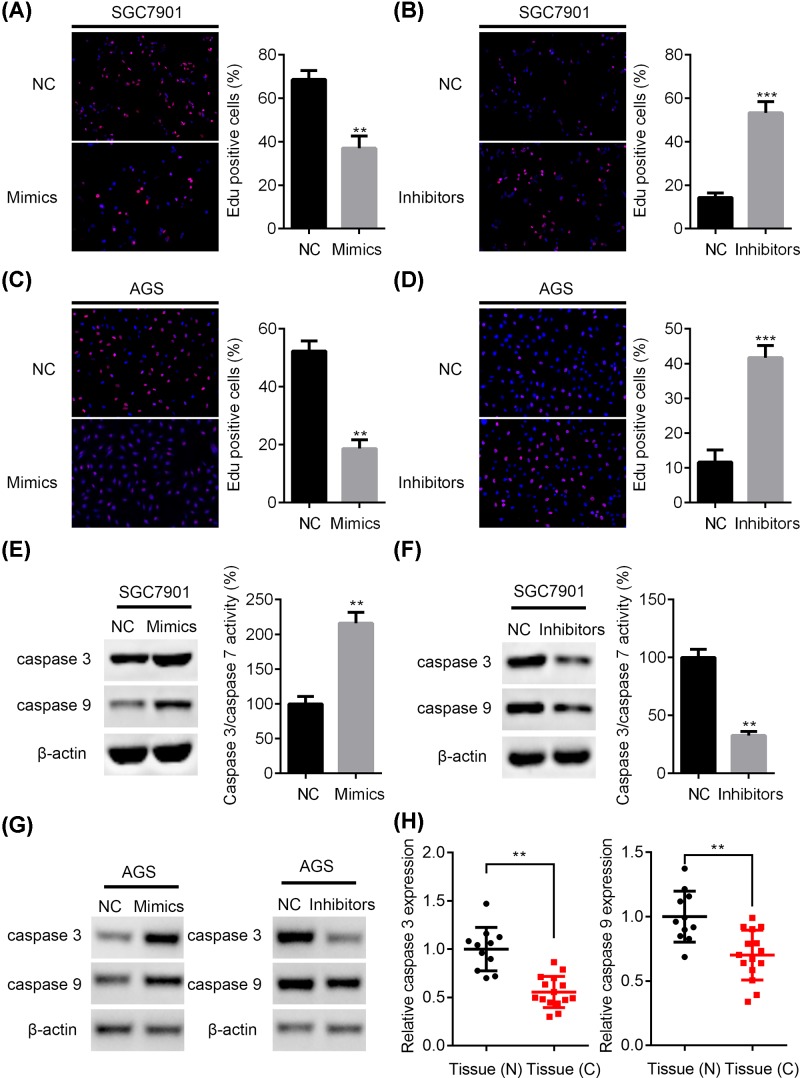
miR-497-5p inhibits EDU cooperation and regulates apoptosis-related gene expression in GC cells (**A,B**) NC or miR-497-5p mimics, NC or miR-497-5p inhibitors transfected SGC7901 cells were subjected to EDU staining. **P<0.01, ***P<0.001. (**C,D**) NC or miR-497-5p mimics, NC or miR-497-5p inhibitors transfected AGS cells were subjected to EDU staining. **P<0.01, ***P<0.001. (**E**) Western blot analysis of caspase 3 and caspase 9, and caspase 3/caspase 7 activity were determined in NC or miR-497-5p mimics transfected SGC7901 cells. **P<0.01. (**F**) Western blot analysis of caspase 3 and caspase 9, and caspase 3/caspase 7 activity were determined in NC or miR-497-5p inhibitors transfected SGC7901 cells. **P<0.01. (**G**) Western blot analysis of caspase 3 and caspase 9 in NC, miR-497-5p mimics and NC, miR-497-5p inhibitors transfected AGS cells. (**H**) qRT-PCR analysis of caspase 3 and caspase 9 in GC (*n* = 15) and normal tissues (*n* = 11). **P<0.01.

EDU is a thymidine analogue for DNA synthesis. Next, we subjected the NC, miR-497-5p over-expressed or silenced SGC7901 and AGS cells for EDU staining. We found that EDU positive cells were decreased in miR-497-5p over-expressed SGC7901 and AGS cells, while they were increased in miR-497-5p silenced cells (Figure 4A–D). In addition, we assessed the effect of miR-497-5p on apoptosis. miR-497-5p mimics led to up-regulation of caspase 3, caspase 9 and increased the activity of caspase 3/caspase 7 (Figure 4E). Opposite results were found in miR-497-5p silenced GC cells (Figure 4F). The expression of caspase 3 and caspase 9 was also increased and decreased in miR-497-5p over-expressed and silenced AGS cells, respectively (Figure 4G). Furthermore, both caspase 3 and 9 were down-regulated in GC tissues comparing with normal tissues (Figure 4H). Our results revealed that miR-497-5p suppressed the GC cell proliferation and growth by promoting DNA synthesis and reducing apoptosis.

### miR-497-5p suppresses cell cycle progression and increases apoptosis in GC cells

**Figure 5:**
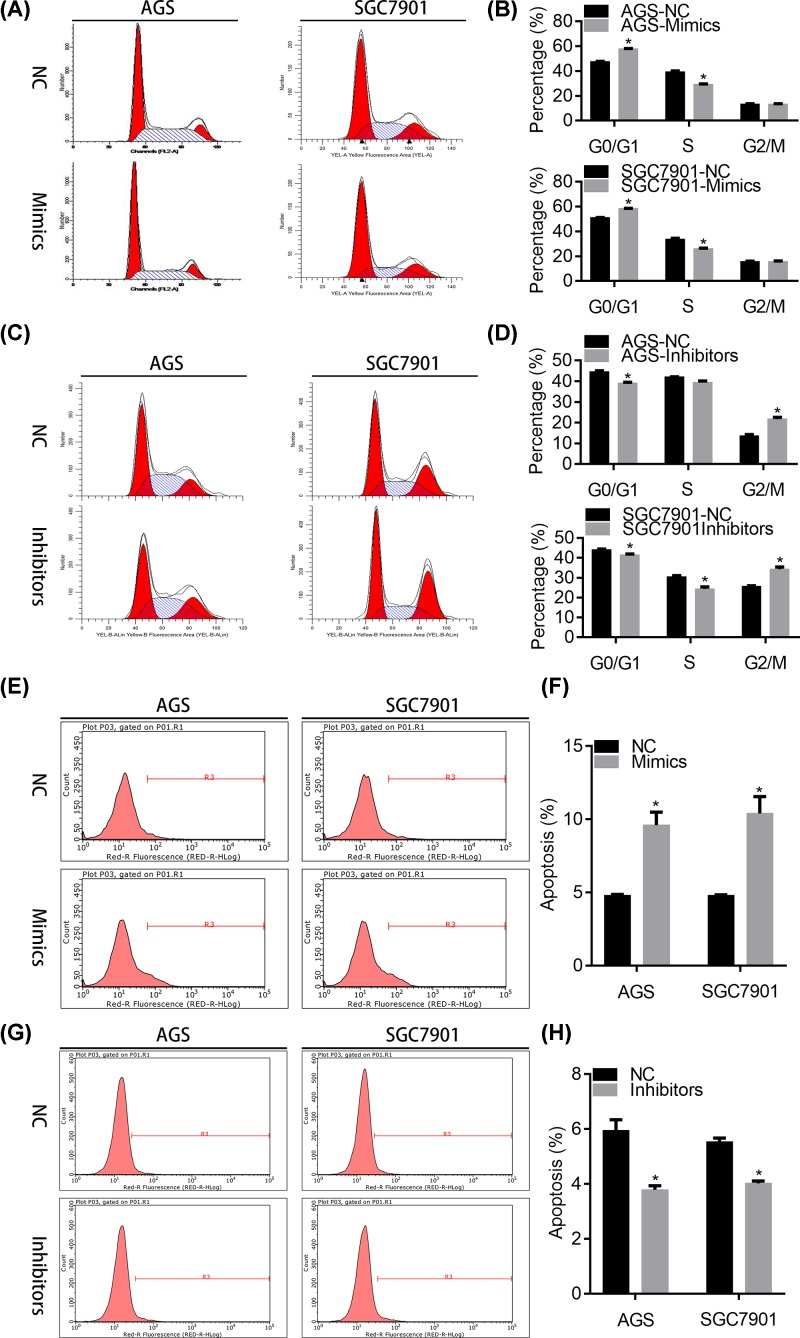
miR-497-5p promotes cell cycle arrest and apoptosis in GC cells (**A,B**) NC or miR-497-5p mimics transfected AGS and SGC7901 cells were subjected to cell cycle analysis. A, representative images. B, quantification results. *P<0.05. (**C,D**) NC or miR-497-5p inhibitors transfected AGS and SGC7901 cells were subjected to cell cycle analysis. A, representative images. B, quantification results. *P<0.05. (**E,F**) NC or miR-497-5p mimics transfected AGS and SGC7901 cells were subjected to apoptosis analysis. A, representative images. B, quantification results. *P<0.05. (**G,H**) NC or miR-497-5p inhibitors transfected AGS and SGC7901 cells were subjected to apoptosis analysis. A, representative images. B, quantification results. *P<0.05.

Enhanced cell cycle progression is a hallmark of cancer cell. We next investigated whether miR-497-5p regulated the cell cycle of GC cells. We found that miR-497-5p over-expression led to increased cell cycle distribution at the G0/G1 phase (Figure 5A,B). The number of GC cells at S phase was reduced in miR-497-5p mimics transfected GC cells (Figure 5A,B). MiR-497-5p inhibitors resulted in decreased G0/G1 phase (Figure 5C,D). G2/M, but not S phase, was increased in miR-497-5p inhibitors transfected GC cells (Figure 5C,D). Moreover, the apoptosis was increased and decreased in miR-497-5p over-expressed and silenced GC cells, respectively (Figure 5E–H). Collectively, miR-497-5p participates in cell cycle and apoptosis of GC cells.

### miR-497-5p inhibits the proliferation of GC cells by targeting PDK3

**Figure 6:**
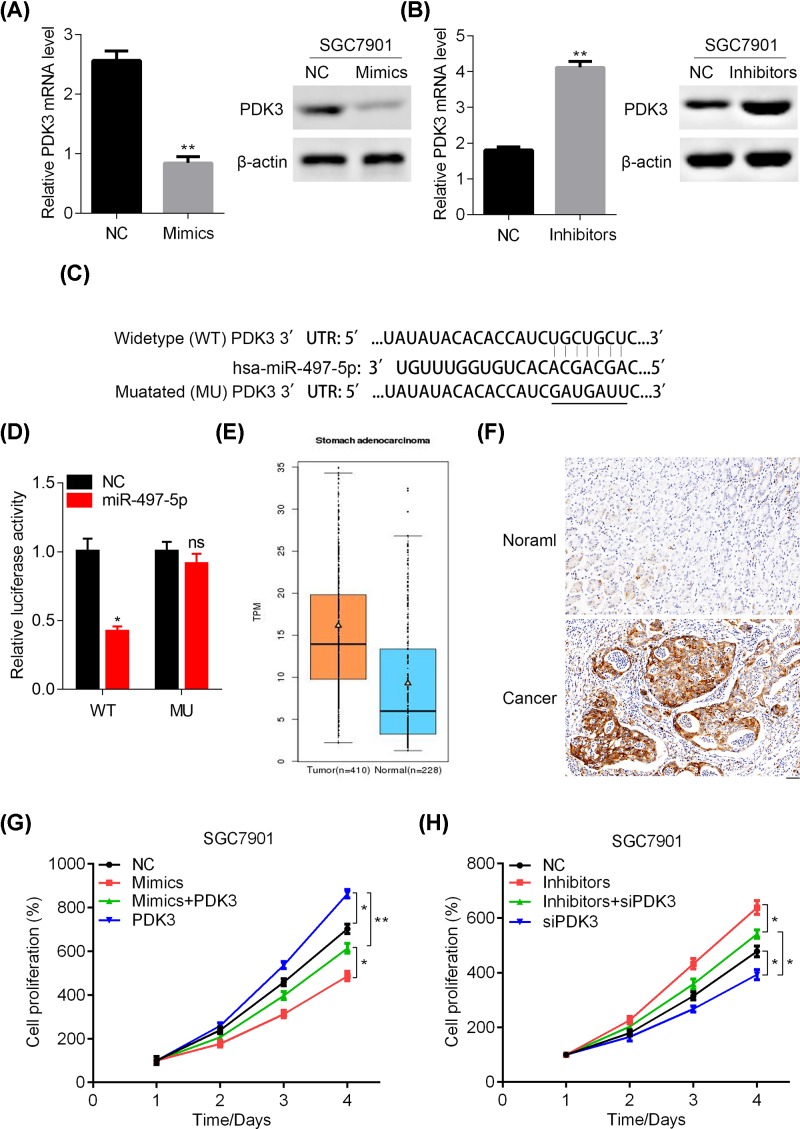
miR-497-5p directly targets PDK3 in GC cells (**A**) A putative miR-497-5p binding site in the PDK3 3′UTR region that was predicted using TargetScan. (**B**) NC or miR-497-5p mimics transfected SGC7901 cells were subjected to qRT-PCR and Western blot analysis of PDK3. **P<0.01. (**C**) NC or miR-497-5p inhibitors transfected SGC7901 cells were subjected to qRT-PCR and Western blot analysis of PDK3. **P<0.01. (**D**) The psiCHECK reporters containing the wild-type (WT) or mutated (MU) 3′UTR of PDK3 gene were co-transfected with miR-497-5p mimics or NC into SGC7901 cells. Luciferase activity was measured at 48 h after transfection. (**E**) mRNA expression of PDK3 in stomach adenocarcinoma (*n* = 410) and normal tissues (*n* = 228). *P*<0.001. (**F**) SGC7901 cells that were transfected with NC, miR-497-5p mimics, miR-497-5p mimics and PDK3 over-expression lentivirus, and PDK3 over-expression lentivirus were subjected to CCK analysis of cell proliferation. *P<0.05, **P<0.01. (**G**) SGC7901 cells that were transfected with NC, miR-497-5p mimics, miR-497-5p mimics and PDK3 over-expression lentivirus, and PDK3 over-expression lentivirus were subjected to CCK analysis of cell proliferation. *P<0.05, **P<0.01. (**H**) SGC7901 cells that were transfected with NC, miR-497-5p inhibitors, miR-497-5p inhibitors and PDK3 knockdown lentivirus, and PDK3 knockdown lentivirus were subjected to CCK analysis of cell proliferation. *P<0.05.

Next, we attempted to elucidate the molecular mechanism by which miR-497-5p inhibited the tumor cell growth and proliferation. Online program TargetScan was used to predict the potential target of miR-497-5p. The information indicates that miR-497-5p binds to the 3′UTR of numerous genes, including PDK3 (Figure 6A). Because PDK3 is an important regulator of glycolysis, which is hyper-activated in cancer development, we examined whether miR-497-5p directly regulated PDK3 in GC cells. qRT-PCR and Western blot assays showed that miR-497-5p up-regulation and down-regulation suppressed and promoted the mRNA and protein abundance of PDK3 in SGC7901 cells, respectively (Figure 6B,C). To test the interaction specificity between miR-497-5p and the 3′UTR of PDK3, we cloned the widetype and mutant 3′UTR sequence to the downstream of the Renilla luciferase reporter gene and measured the effect of miR-497-5p on reducing luciferase expression. The miR-497-5p reduced the reported gene expression in PDK3 WT but not in MU expressed cells (Figure 6D). We also found that PDK3 was highly expressed in GC tissues based on the TCGA database and our immunohistochemical staining (Figure 6E,F). Importantly, PDK3 over-expression reversed the inhibitory effect of miR-497-5p mimics on SGC7901 cells proliferation, while PDK3 knockdown reduced the viability of miR-497-5p down-regulated SGC7901 cells (Figure 6G,H). Taken together, miR-497-5p directly targeted PDK3 to suppress the GC cell proliferation and growth.

## Discussion

In the past few decades, miRNAs have been demonstrated to play important roles in cancer development, either by acting as oncogenes or tumor suppressors [17–20]. Here, we investigated the involvement of miR-497-5p in gastric cancer. miR-497-5p was down-regulated in gastric cancer tissues and its abundance was much lower in the cancer tissues of high-stage GC. *In vitro* proliferation and growth assays demonstrated the tumor suppressive role of miR-497-5p in GC. DNA synthesis and cell cycle were inhibited, while apoptosis was induced by miR-497-5p. At the molecular level, PDK3 oncogene was a direct target for miR-497-5p.

miR-497-5p, which belongs to the miR-15/107 group, harbors the seed sequence AGCAGC that is an essential determinant of target recognition [21]. Several studies have demonstrated that miR-497-5p is closely associated with cancer development [22,23]. Here, we observed that miR-497-5p expression was reduced in gastric cancer tissues as comparing with the normal tissues. TCGA database was consistent with our results. Furthermore, the cancer tissues of stage TMNIV exhibited lower miR-497-5p level than those of stage TMNII. There was also an inverse relationship between miR-497-5p expression and PCNA expression, which was always highly expressed in cancer tissues. It has been reported that miR-497-5p ectopic expression suppresses, while its knockdown enhances the growth of different cancers, such as melanoma [6], non-small-cell lung cancer [24] and cervical cancer [23]. In the present study, we found that miR-497-5p over-expression inhibited the proliferation and growth of GC cells, while its knockdown enhanced GC cell viability. The EDU cooperation, which represents a symbol of DNA synthesis, was suppressed by miR-497-5p. Some studies have shown that microRNAs regulate the apoptosis and cell cycle progression of GC cells. For example, miR-876-5p negatively regulates the apoptosis of GC cells [25]. MiR-383 suppresses the cell cycle process in GC cells through targeting Cyclin E2 [26]. Here, we observed that the apoptosis was increased by miR-497-5p mimics, while opposite results were observed in miR-497-5p silenced cells. Furthermore, the cell cycle progression was arrested by miR-497-5p at G0/G1 phase in GC cells. We predicted that miR-497-5p served as a tumor suppressive microRNA in gastric cancer.

The PDK family contains four members, including PDK1, PDK2, PDK3 and PDK4. Tissue specific expression of these kinases shows that they may have different physiological and pathological functions [27,28]. For instance, PDK4 is involved in metabolic changes under high-fat diet condition and diabetes [29,30], while PDK1 and PDK3 are more likely to contribute to metabolic switch and cell survival under hypoxia [9–11]. On the other hand, PDK3 plays an important role in carcinogenesis. Over-expression of PDK3 is correlated with poor prognosis of acute myeloid leukemia [13]. Silencing of PDK3 induces the cell death of glioblastoma multiforme [14]. In melanoma, inhibition of PDK3 activity by dichloroacetate enhances the antitumor effects of elesclomol [31]. These findings suggest that PDK3 is a promising target for cancers. However, the function of PDK3 in gastric cancer is unclear. Here, we found that PDK3 was a direct target for miR-497-5p. miR-497-5p was up-regulated and down-regulated in miR-497-5p silenced and over-expressed gastric cancer cells, respectively. Luciferase reporter experiments identified that miR-497-5p bind to the 3′UTR region of PDK3. Mutation in the 3′UTR of PDK3 had no effect of miR-497-5p on target suppression. Importantly, PDK3 was highly expressed in gastric cancer tissues based on the TCGA database and our immunohistochemical staining. Over-expression of PDK3 reversed the suppressive effect of miR-497-5p on gastric cancer cell proliferation. PDK3 knockdown reduced the viability of gastric cancer cells with silenced miR-497-5p. Our results indicated that PDK3 was the downstream target for miR-497-5p and a potential oncogene in gastric cancer.

In summary, we provided for the first time that miR-497-5p functioned as a tumor suppressive microRNA in gastric cancer. miR-497-5p was down-regulated in gastric cancer tissues and it suppressed the proliferation and growth of gastric cancer cells. Mechanistically, miR-497-5p directly targeted PDK3 at its 3′UTR region. Our study proposed the miR-497-5p/PDK3 as a potential therapeutic target for gastric cancer.

## Abbreviations

GC: gastric cancer
miRNA: microRNA
PDK3: pyruvate dehydrogenase kinase 3
qRT-PCR: quantitative real-time PCR
